# Supplementary material for: Osmotic disruption of chromatin induces Topoisomerase 2 activity at sites of transcriptional stress
Source: Nat Commun. 2024 Dec 5;15:10606. doi: 10.1038/s41467-024-54567-6 (PMC11621772; doi:10.1038/s41467-024-54567-6)
Supplement: Supplementary file 1 — Supplementary Information [file 41467_2024_54567_MOESM1_ESM.pdf]

# Osmotic disruption of chromatin induces Topoisomerase 2 activity at sites of transcriptional stress

William H. Gittens, Rachal M. Allison, Ellie M. Wright, George G. B. Brown and Matthew J. Neale

Genome Damage and Stability Centre, School of Life Sciences, University of Sussex, Brighton, UK.

Correspondence: w.gittens@sussex.ac.uk; m.neale@sussex.ac.uk

## Supplementary Figures 1-10

## Supplementary Tables 1-3

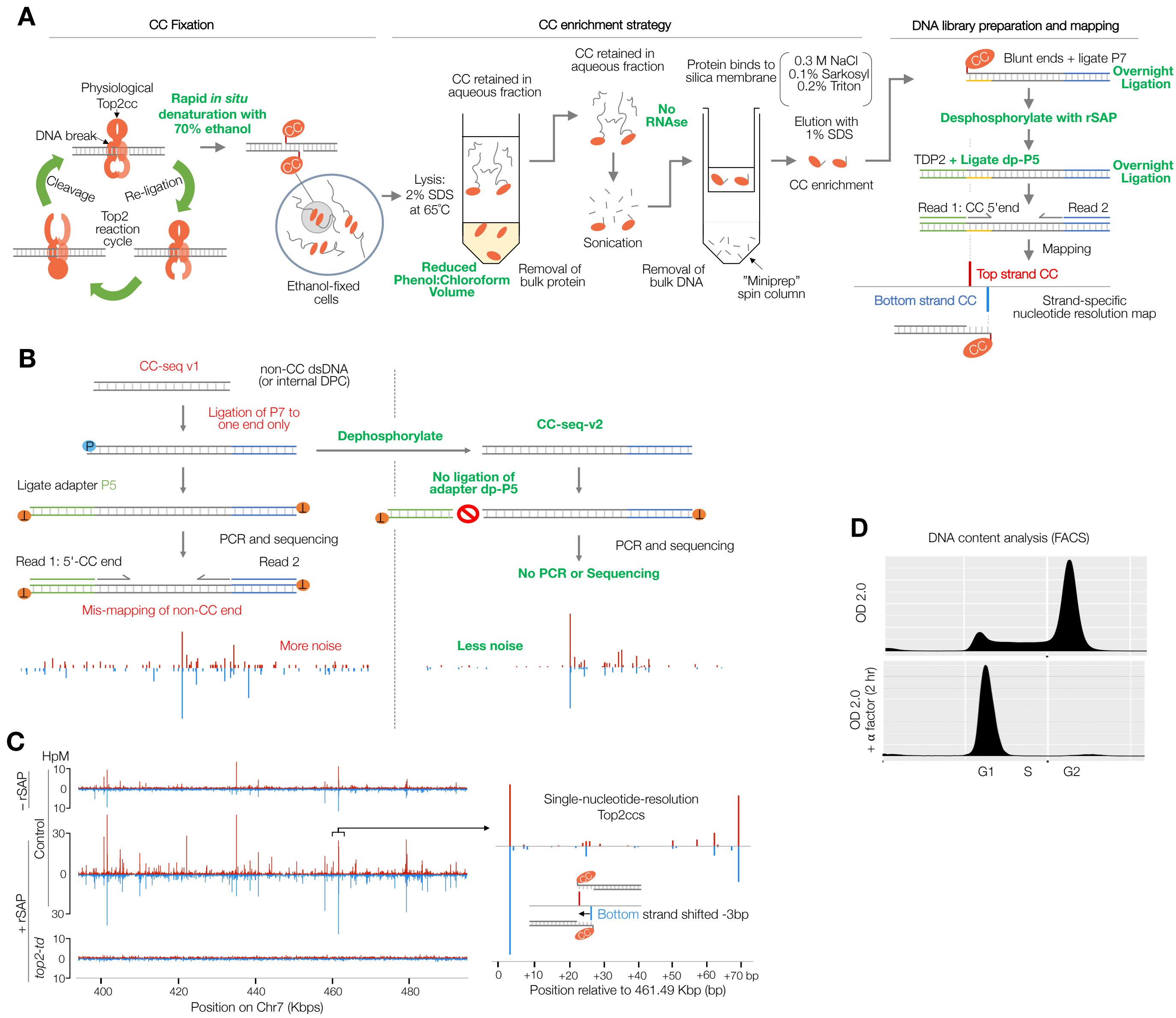


**Supplementary Figure 1. An updated CC-seq-v2 method dramatically increases signal/noise to enable mapping of physiological Top2 activity. (A)** Schematic summary of the CC-seq-v2 method. Modifications to the original v1 method are indicated in green text. **(B)** Schematic explanation of how our new dephosphorylation strategy reduces background noise in CC-seq-v2. **(C)** Comparison of CC-seq v2 maps of physiological Top2ccs generated without (top) and with (middle and bottom) the new dephosphorylation strategy, in G1-arrested control (top and middle) and *top2-td* (bottom) W303 *S. cerevisiae* cells, under degron inducing conditions. Stronger peaks in middle vs top track are due to increased sensitivity via the use of the dephosphorylation strategy. Loss of peaks in bottom vs middle track is due to specificity of these signals for Top2 activity. The right hand panel is zoomed in view of a 70 bp region, illustrating top and bottom strand parity at single nucleotide resolution following displacement of the bottom strand signals by -3 bp. **(D)** Flow cytometry analysis of cellular DNA content in asynchronously growing yeast (top), and yeast arrested in G1 via addition of alpha mating factor for 2 hours.

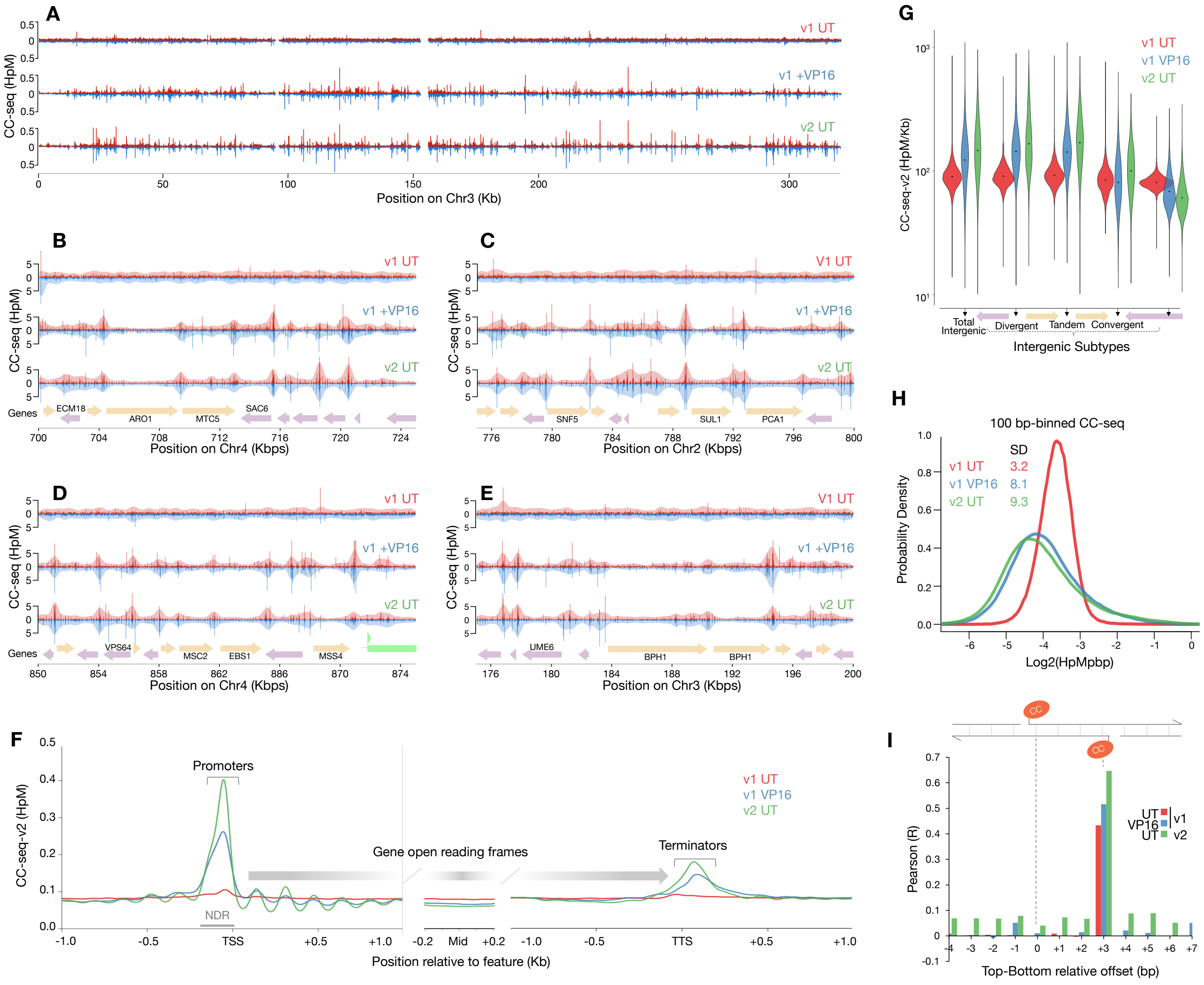


**Supplementary Figure 2. Comparison of CC-seq-v1 and CC-seq-v2. (A)** 100 bp binned CC-seq maps of Top2ccs across chromosome 3, either generated previously[1] using CC-seq-v1 in untreated and VP16-treated asynchronous *S. cerevisiae*, or generated herein using CC-seq-v2 in G1-arrested *S. cerevisiae.* **(B–E)** Comparison of unbinned CC-seq-v1 and v2 data, over 25 kb range at the indicated loci. Red and blue traces indicate Top2-linked 5′ DNA termini on the top and bottom strands, respectively. In each track, the pale red and blue curves are the same data smoothed with a sliding 1 kb Hann window. Coloured arrows in the bottom panel indicate positions and orientation of specific genomic elements. **(F)** Average (pileup) of CC-seq-v1 and v2 Top2cc signals around TSSs (left), gene body midpoints (middle), and TTSs (right). Signals were smoothed with a 100 bp sliding Hann window. (**G)** Violin plots of genome-wide CC-seq-v1 and v2 Top2cc signal densities in specific genome compartments. **(H)** Density plot of 100 bp binned CC-seq-v1 and v2 data, with inset standard deviations (SD) indicating highest dynamic range in the untreated CC-seq-v2 map. **(I)** Pearson correlation (R) of CC-seq-v1 and v2 signals on top and bottom strands, after displacement (Top-Bottom) by the indicated distance. Positive values indicate bottom strand displacements leftwards relative to the top strand. Source data are provided as a Source Data file.

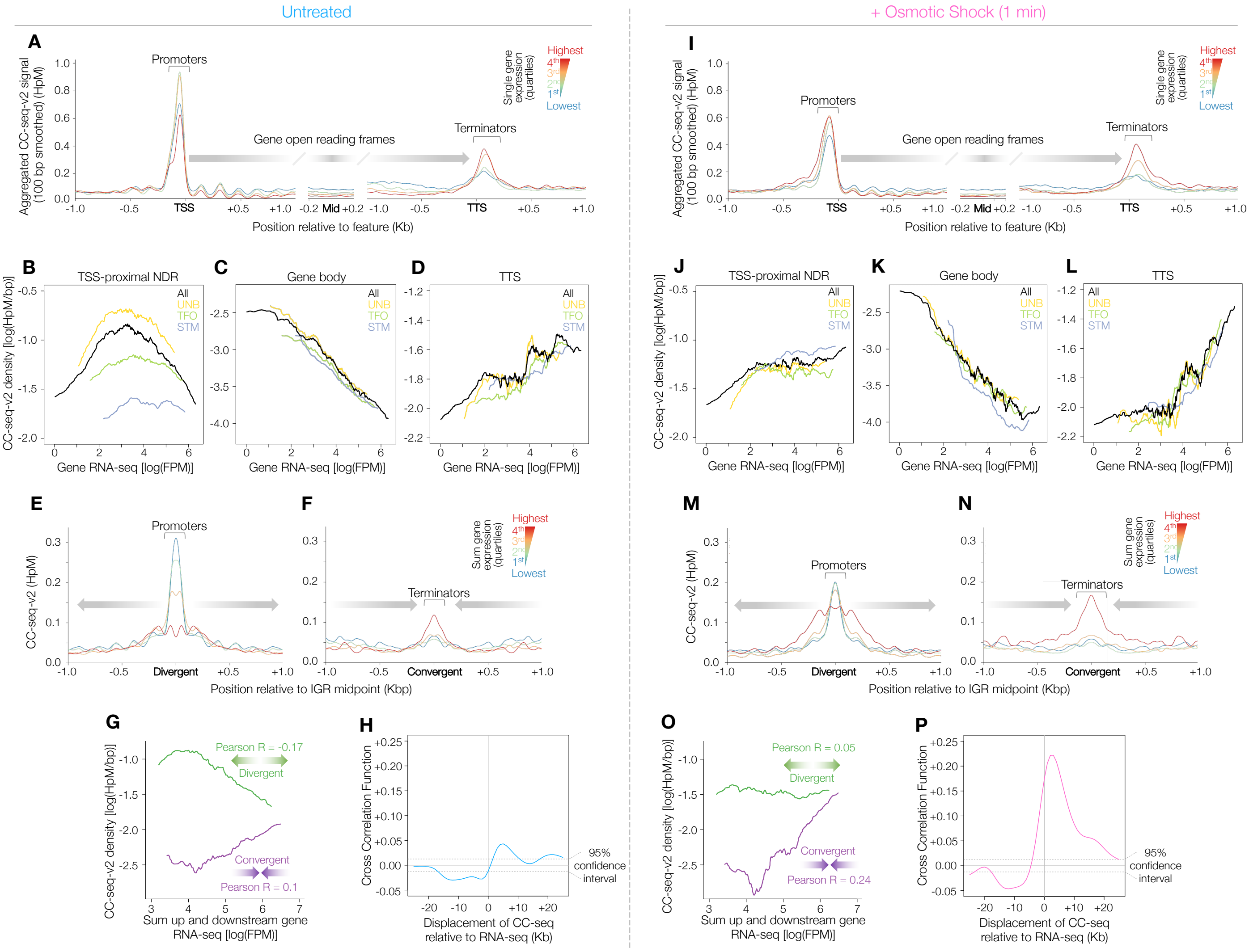


**Supplementary Figure 3. Quantitative relationships between Top2 activity and local transcription in unperturbed cells and following transient osmotic shock. (A)** Average (pileup) of CC-seq-v2 Top2cc signals around TSSs (left), gene body midpoints (middle), and TTSs (right), stratified into four levels of transcriptional activity measured by RNA-seq. Signals were smoothed with a 100 bp sliding Hann window. **(B–D)** Gene transcriptional activity measured by RNA-seq is plotted against CC-seq-v2 Top2cc signal in the NDR (B), the gene body (C), and a 300 bp window immediately downstream of the transcription termination site (TTS) (D). The analysis was performed separately for all genes (black line; 500 gene sliding average), STM, TFO and UNB genes (coloured lines; 300 gene sliding average). The analysis was not performed for RP genes, because there are too few for the sliding average to be effective. **(E–F)** Average (pileup) of CC-seq-v2 Top2cc signals around divergent (E) and convergent (F) intergenic region (IGR) midpoints, stratified into four levels of sum neighbouring gene transcriptional activity measured by RNA-seq. Signals were smoothed with a 100 bp sliding Hann window. **(G)** Sum neighbouring gene transcriptional activity measured by RNA-seq is plotted against CC-seq-v2 Top2cc signal in the IGR (250 gene sliding average). The analysis was performed separately for convergent and divergent IGRs (coloured lines). **(H)** Cross-correlation function (CCF) of 1 kb-binned stranded CC-seq-v2 Top2cc and RNA-seq transcription signals over displacements of -25 to +25 kb. The arrow indicates the 5'→ 3' direction of transcription. At positive displacements, RNA-seq signals are correlated with 3'-wards CC-seq signals. Dotted lines indicate the 95% confidence interval. **(I-P)** As described for panels (A-H) but in cells subjected to 1 minute of osmotic shock. Source data are provided as a Source Data file.

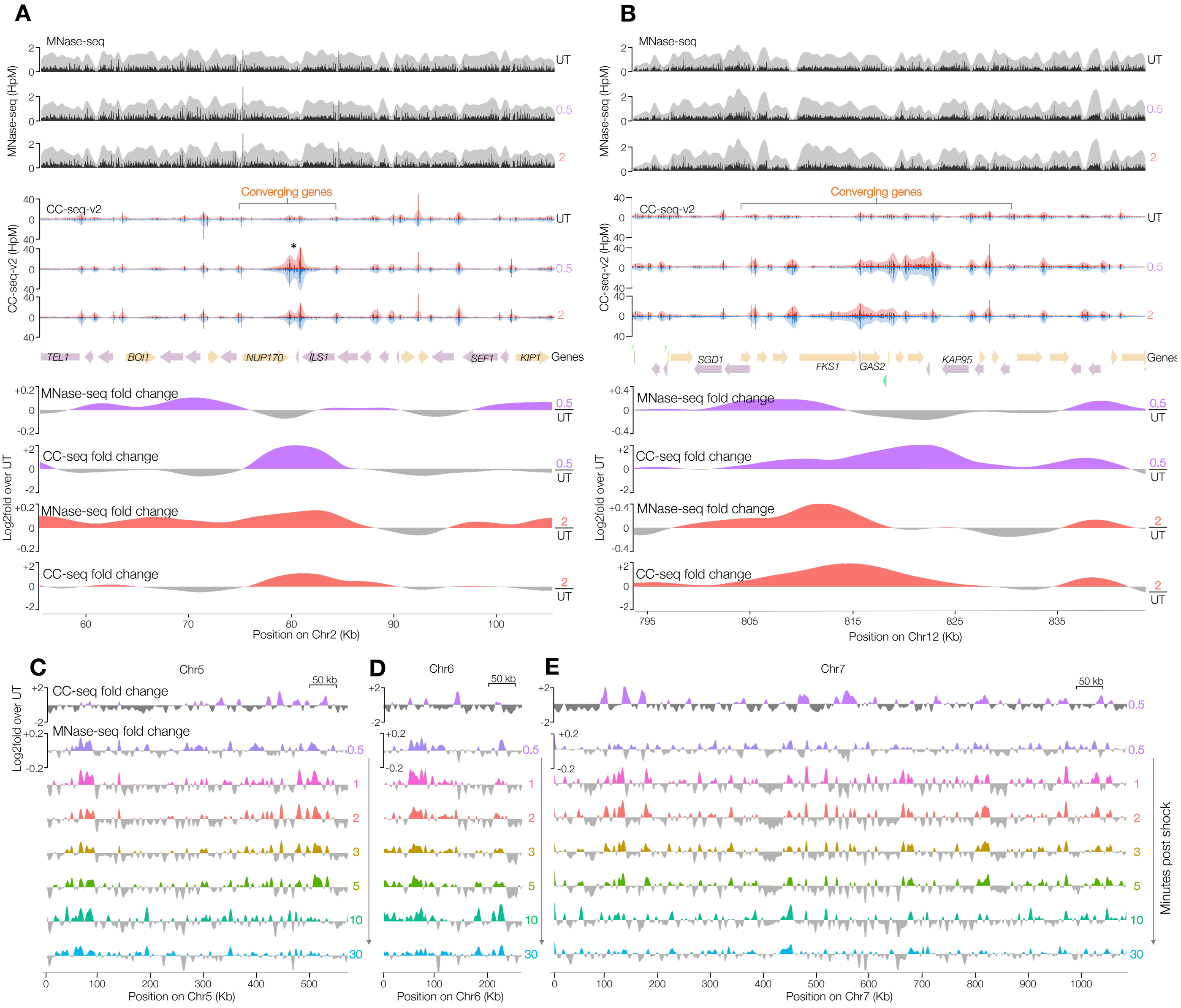


**Supplementary Figure 4. Quantitative relationships between Top2 activity and local transcription in unperturbed cells and following transient osmotic shock. (A,B)** Comparison of MNase-seq maps of nucleosome occupancy (tracks 1–3) and 10 bp binned CC-seq-v2 maps of physiological Top2ccs (tracks 4–6) in G1-arrested *S. cerevisiae*, over 50 kb range at the indicated loci in untreated cells (UT) and at the indicated minutes after osmotic shock with 0.6 M sorbitol. In tracks 1–3, black traces indicate MNase-seq fragment midpoints. Pale curves are the same data smoothed with a 1 kb Hann window. In tracks 4–6, red and blue traces indicate Top2-linked 5′ DNA termini on the top and bottom strands, respectively. In each track, the pale red and blue curves are the same data smoothed with a sliding 1 kb Hann window. Coloured arrows in the middle panel indicate positions and orientation of specific genomic elements. The lower four tracks are log2 fold change in the MNase-seq or CC-seq-v2 data in minutes following osmotic shock relative to untreated cells (UT), smoothed with a 10 kb Hann window. **(C–E)** Log2 fold change in CC-seq-v2 maps of Top2ccs (track 1) or MNase-seq maps of nucleosome occupancy (tracks 2–8) in minutes following osmotic shock relative to untreated cells (UT) across chromosome 5 (C), 6 (D), and 7 (E), smoothed with a 10 kb Hann window, revealing correlations between changes in chromatin structure and changes in Top2 activity during the 30 minute osmotic shock period (coloured tracks).

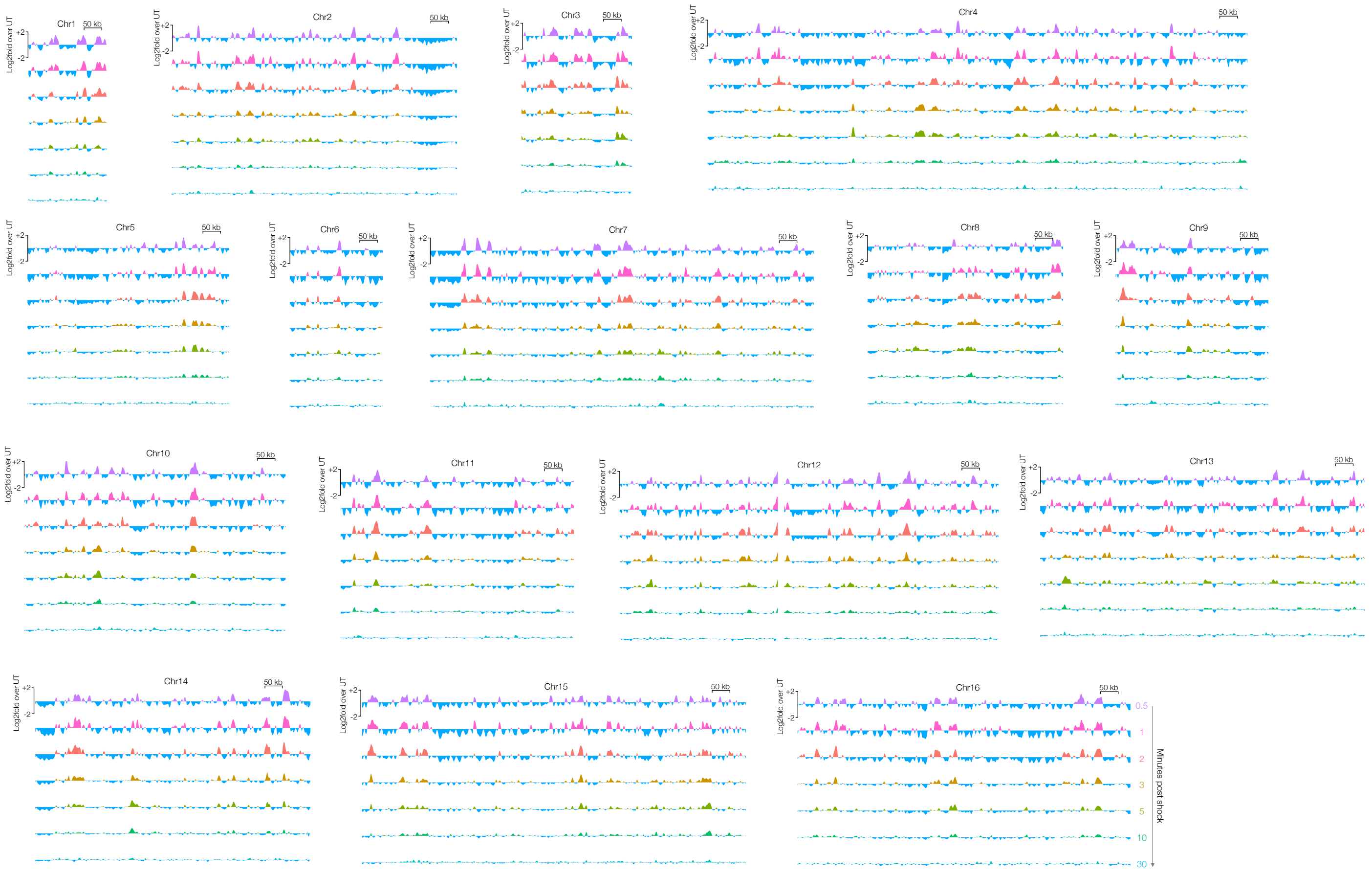


**Supplementary Figure 5. Chromosome-wide redistribution of Top2 activity following acute osmotic shock.** Log2 fold change in CC-seq-v2 maps of Top2ccs in minutes following osmotic shock (0.6 M sorbitol) relative to untreated cells (UT) across each chromosome smoothed with a 10 kb Hann window, revealing regions of relatively greater Top2ccs in each comparison and how this changes during the 30 minute osmotic shock period.

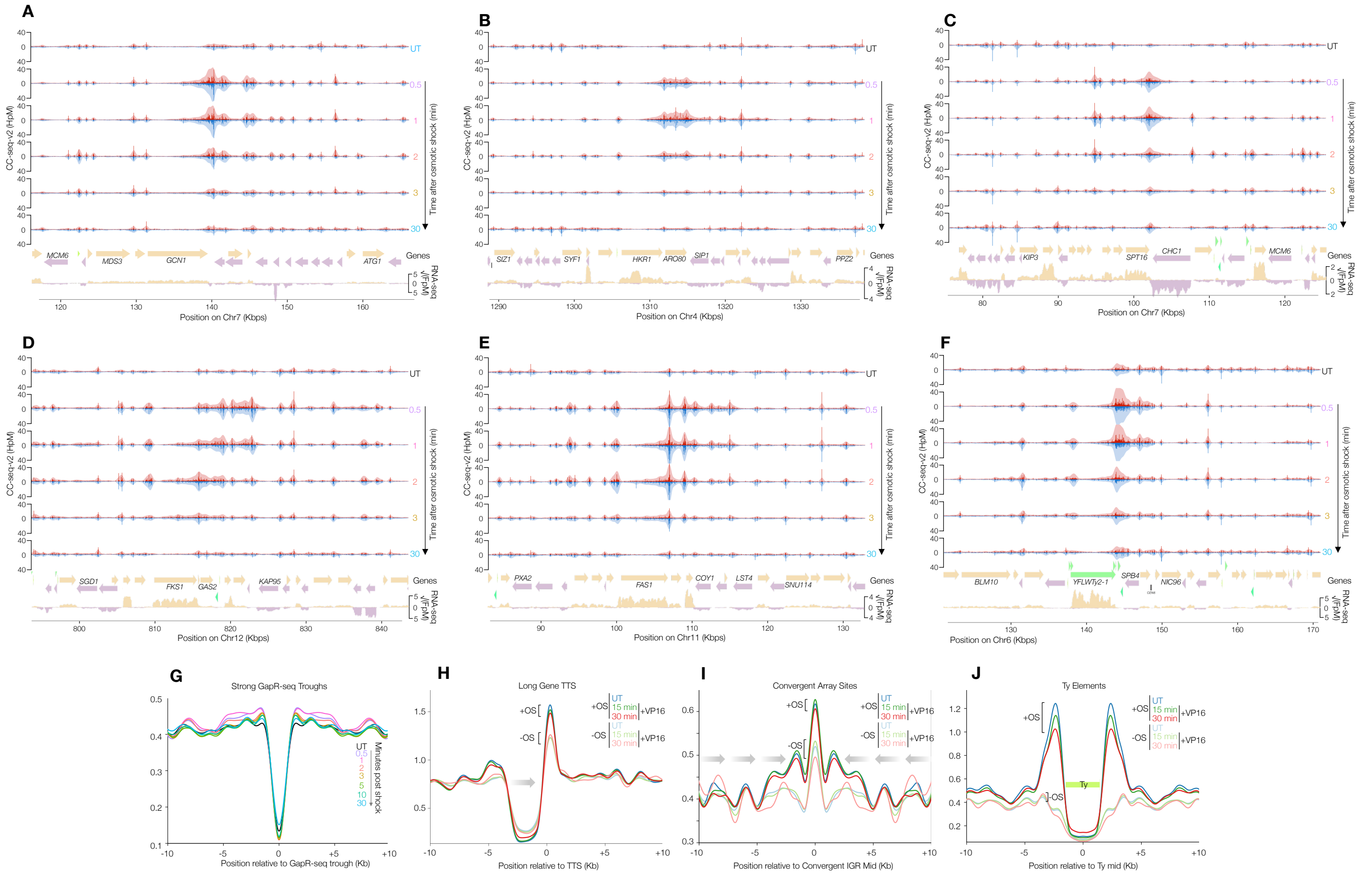


**Supplementary Figure 6. Hotspots of transcription-associated positive superhelical stress. (A-F)** 10 bp binned CC-seq-v2 maps of physiological Top2ccs in G1-arrested *S. cerevisiae*, over 50 kb range at the indicated loci in untreated cells (top) and at the indicated minutes after osmotic shock (0.6M sorbitol). Red and blue traces indicate Top2-linked 5′ DNA termini on the top and bottom strands, respectively. Arrows and rectangles in the bottom panel indicate positions of specific genomic elements, with stranded RNA-seq data from untreated cells plotted below them. In each track, the pale red and blue curves are the same data smoothed with a sliding 1 kb Hann window. **(G)** Average (pileup) of CC-seq-v2 Top2cc signals around troughs of GapR ChIP-seq[2]. Relationship to GapR peaks is presented in Fig 5H. Signals were smoothed with a 2 kb sliding Hann window. **(H-J)** Average (pileup) of CC-seq-v2 signals around long gene transcription termination sites (TTSs) (H), convergent gene arrays (I), and Ty elements (J), respectively, in untreated *pdr1*Δ::*PDR1-DBD-CYC8 S. cerevisiae* cells or following treatment for 15 or 30 min with 100 µM etoposide (VP16). Each untreated, or VP16-treated sample was subsequently subjected (dark colours) or not (light colours) to a 2 min osmotic shock with 0.6 M sorbitol, prior to processing with CC-seq-v2. Data are smoothed with a sliding 2 kb Hann window. The osmotically unshocked and shocked data are indicated by brackets.

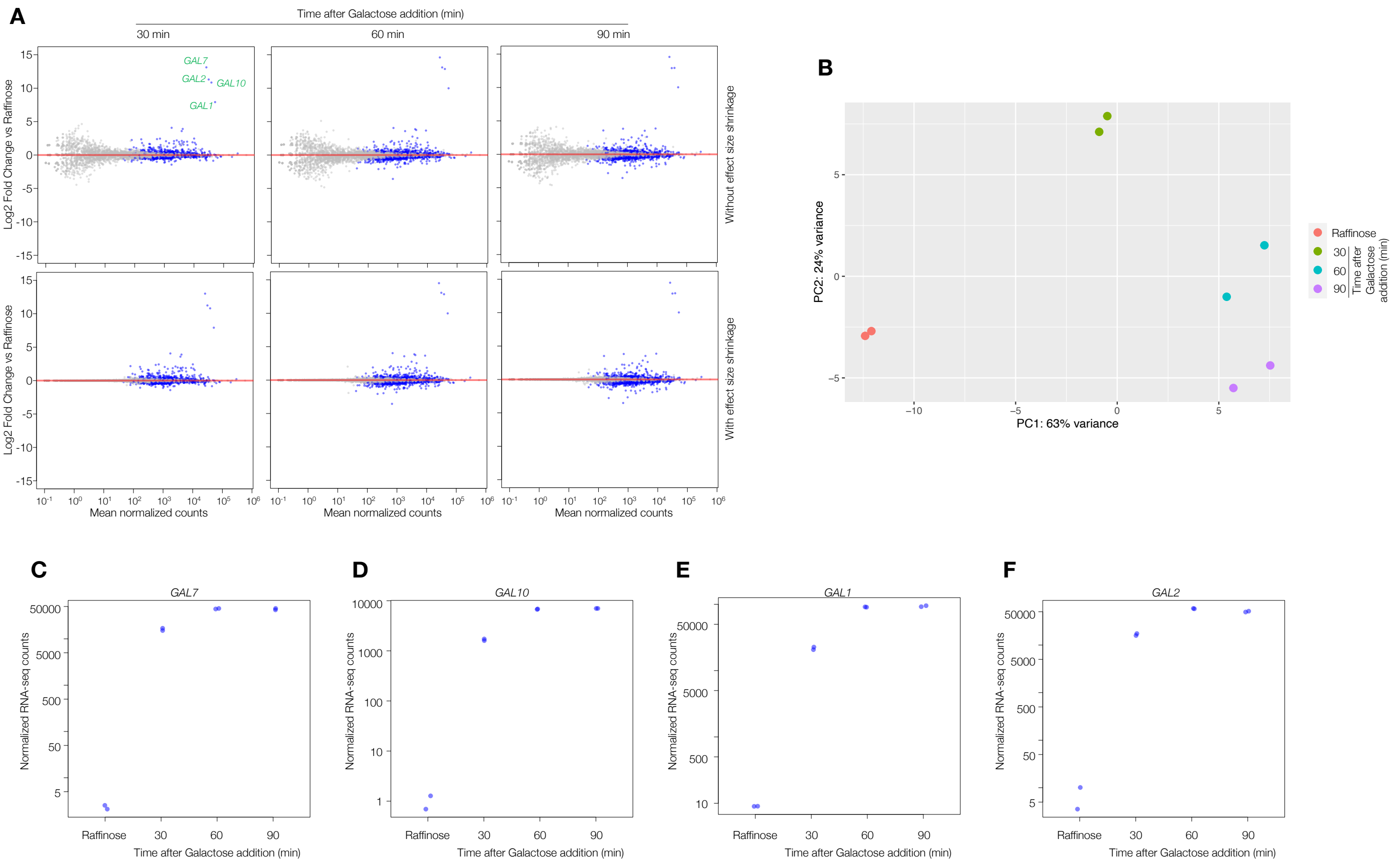


**Supplementary Figure 7. Galactose-induced transcription of *GAL* genes. (A)** MA plots of mean normalised RNA-seq counts against fold change relative to uninduced raffinose cultures, at 30, 60 and 90 min post-induction with galactose. Data are presented before (top) and after (bottom) effect size shrinkage via approximate posterior estimation for generalised linear models (R package *apeglm*). Grey and blue circles indicate nonsignificant and significant genes (negative binomial GLM two-sided likelihood ratio test FDR-adjusted $P$-value < 0.05), respectively. **(B)** Principal component analysis (PCA) of RNA-seq data. Coloured dots indicate individual replicate data. **(C-F) N**ormalised RNA-seq counts for *GAL7, GAL10, GAL1* and *GAL2*, over the time course of galactose induction. Data were processed and plotted with the *R* package *DEseq2*. Individual points indicate individual replicates. Source data are provided as a Source Data file.

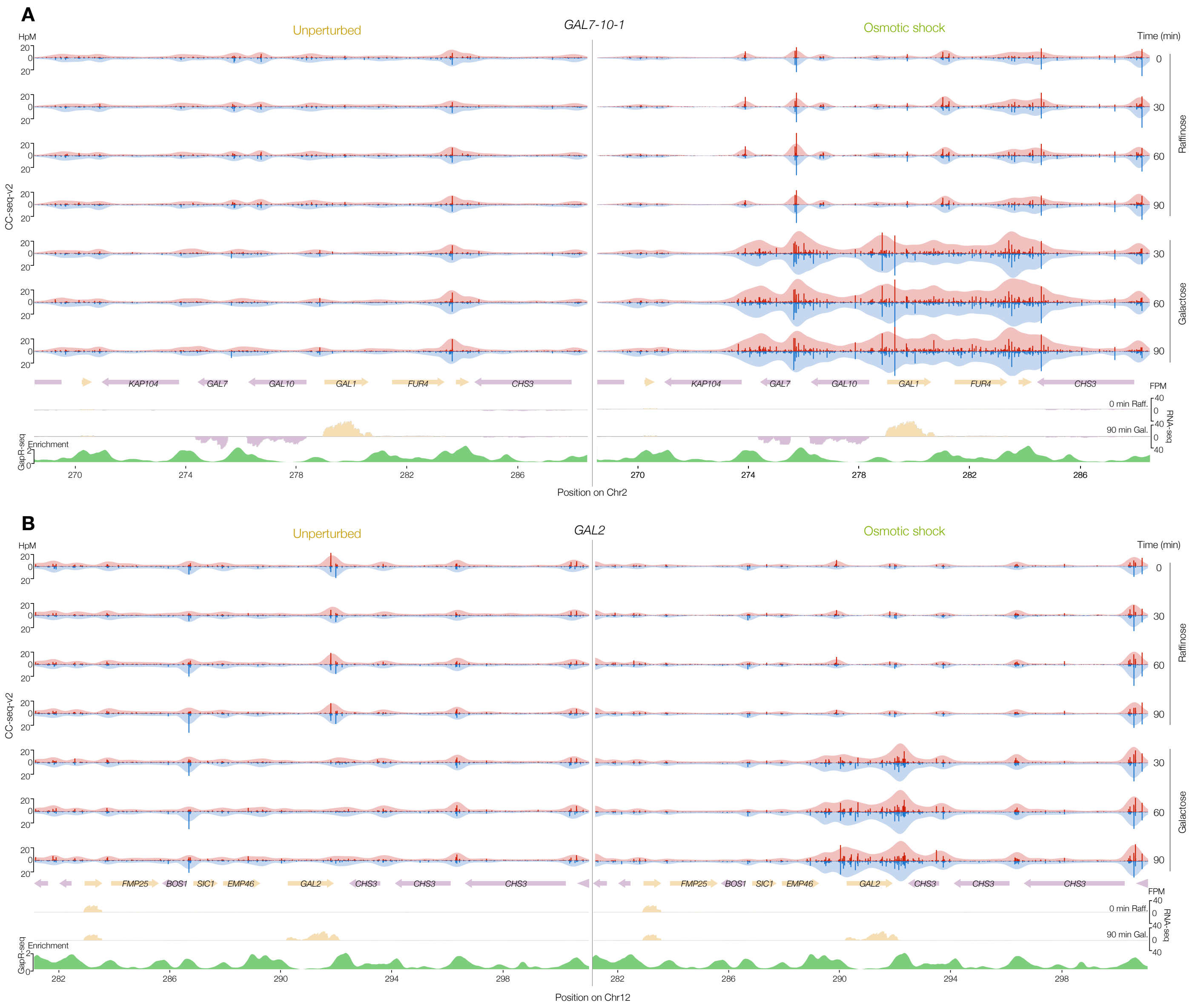


**Supplementary Figure 8. Individual CC-seq-v2 timepoints, at *GAL7-10-1* and *GAL2* loci throughout galactose induction. (A-B)** Nucleotide-resolution CC-seq-v2 maps of physiological Top2ccs in G1-arrested *S. cerevisiae*, over 10 kb range at the *GAL1-10* (A) and *GAL2* (B) loci before (top four tracks) and after (lower three tracks) shifting from raffinose-containing media to galactose-containing media in untreated cells (left) or after subjecting cells to sorbitol-induced osmotic shock for 2 minutes (right). Red and blue traces indicate Top2-linked 5′ DNA termini on the top and bottom strands, respectively. Arrows and rectangles in the bottom panel indicate positions of specific genomic elements with labels. In each track, the pale red and blue curves are the same data smoothed with a sliding 1 kb Hann window. Stranded RNA-seq data from cells induced for 90 min with galactose is plotted in pink and yellow. A GapR-ChIP enrichment map of positive superhelicity is plotted in green[2].

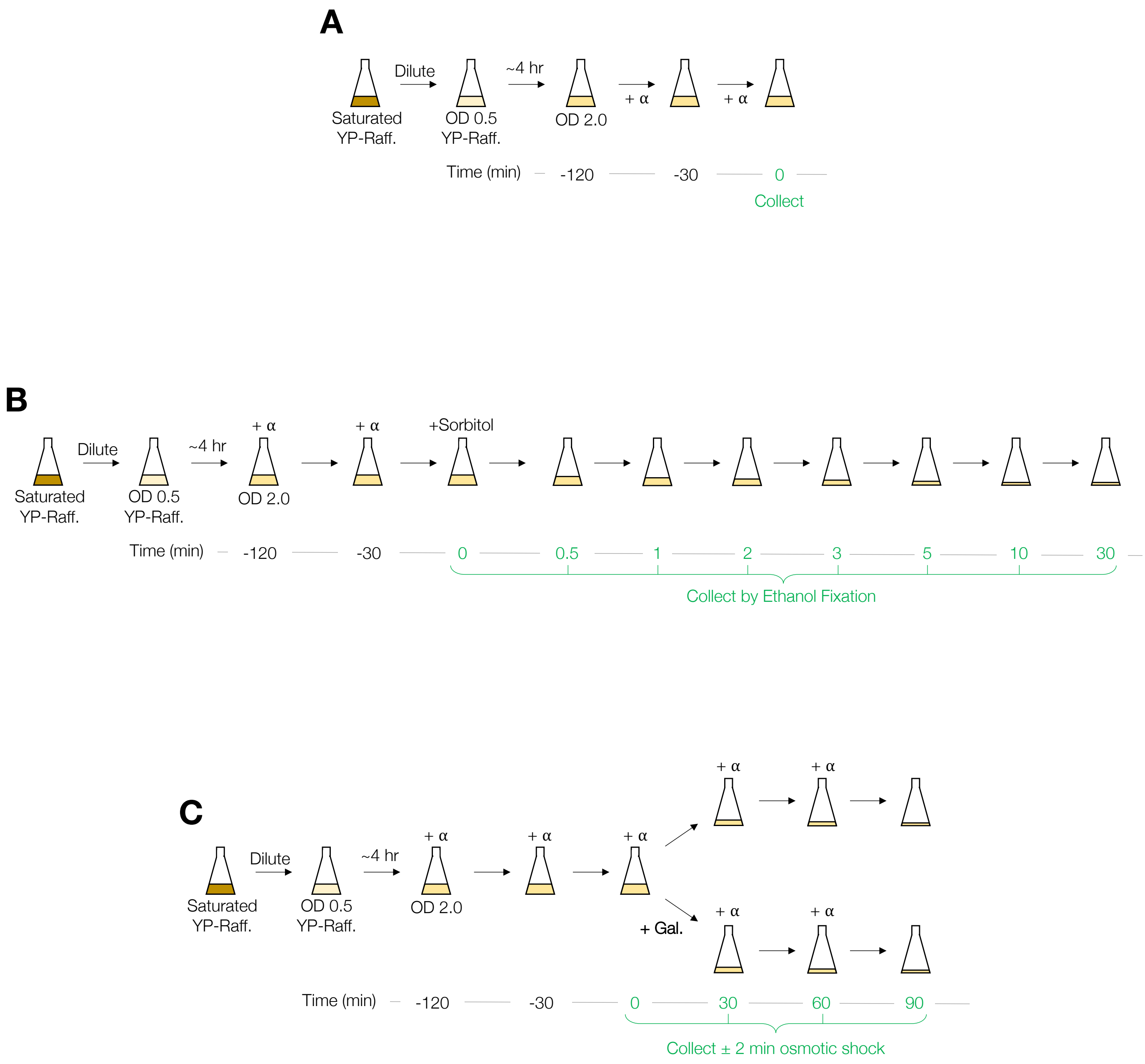


**Supplementary Figure 9. Diagrammatic experimental designs employed in this study. (A)** G1-arrested cells were grown exponentially in YP-raffinose from OD600=0.5 to 2.0 and treated sequentially with the alpha factor mating pheromone 120 minutes, then again at 30 minutes prior to harvesting. **(B)** For osmotic shock timecourses, G1-arrested cells were prepared as in A, but mixed at 0 minutes with a final concentration of 0.6M sorbitol, then samples immediately fixed at the indicated time intervals by adding ethanol (–20˚C) to a final concentration of 70%. **(C)** For galactose-induction experiments, G1-arrested cells were prepared as in A, but split at 0 minutes with half of the culture mixed with a final concentration of 2% galactose, then samples removed at indicated time intervals, subjected to a 2 minute sorbitol-induced osmotic shock, then fixed by adding ethanol (–20˚C) to a final concentration of 70%. Additional alpha factor was added at 30 minutes and 60 minutes to counteract potential proteolysis in the media and prevent cells exiting G1 phase.

**A**

| | WG223b_sWG1_Sorbitol_UT_rep1 | WG224b_sWG1_Sorbitol_30s_rep1 | WG225b_sWG1_Sorbitol_1min_rep1 | WG226b_sWG1_Sorbitol_2min_rep1 | WG227b_sWG1_Sorbitol_3min_rep1 | WG228b_sWG1_Sorbitol_5min_rep1 | WG229b_sWG1_Sorbitol_10min_rep1 | WG239-sWG1-Sorbitol-UT-rep2 | WG240-sWG1-Sorbitol-30s-rep2 | WG241-sWG1-Sorbitol-1min-rep2 | WG242-sWG1-Sorbitol-2min-rep2 | WG243-sWG1-Sorbitol-3min-rep2 | WG244-sWG1-Sorbitol-5min-rep2 | WG245-sWG1-Sorbitol-10min-rep2 | WG246-sWG1-Sorbitol-30min-rep2 |
|---|---|---|---|---|---|---|---|---|---|---|---|---|---|---|---|
| WG223b_sWG1_Sorbitol_UT_rep1 | 1.00 | 0.61 | 0.57 | 0.64 | 0.76 | 0.85 | 0.89 | 0.97 | 0.71 | 0.65 | 0.78 | 0.85 | 0.88 | 0.90 | 0.92 |
| WG224b_sWG1_Sorbitol_30s_rep1 | 0.61 | 1.00 | 0.94 | 0.87 | 0.81 | 0.75 | 0.69 | 0.60 | 0.90 | 0.90 | 0.79 | 0.75 | 0.70 | 0.65 | 0.61 |
| WG225b_sWG1_Sorbitol_1min_rep1 | 0.57 | 0.94 | 1.00 | 0.91 | 0.82 | 0.73 | 0.67 | 0.57 | 0.86 | 0.91 | 0.79 | 0.73 | 0.68 | 0.63 | 0.58 |
| WG226b_sWG1_Sorbitol_2min_rep1 | 0.64 | 0.87 | 0.91 | 1.00 | 0.91 | 0.82 | 0.75 | 0.63 | 0.88 | 0.92 | 0.89 | 0.82 | 0.76 | 0.70 | 0.63 |
| WG227b_sWG1_Sorbitol_3min_rep1 | 0.76 | 0.81 | 0.82 | 0.91 | 1.00 | 0.92 | 0.86 | 0.75 | 0.86 | 0.86 | 0.93 | 0.91 | 0.86 | 0.82 | 0.76 |
| WG228b_sWG1_Sorbitol_5min_rep1 | 0.85 | 0.75 | 0.73 | 0.82 | 0.92 | 1.00 | 0.95 | 0.84 | 0.82 | 0.79 | 0.90 | 0.95 | 0.95 | 0.91 | 0.84 |
| WG229b_sWG1_Sorbitol_10min_rep1 | 0.89 | 0.69 | 0.67 | 0.75 | 0.86 | 0.95 | 1.00 | 0.88 | 0.79 | 0.74 | 0.87 | 0.93 | 0.96 | 0.96 | 0.90 |
| WG239-sWG1-Sorbitol-UT-rep2 | 0.97 | 0.60 | 0.57 | 0.63 | 0.75 | 0.84 | 0.88 | 1.00 | 0.72 | 0.65 | 0.79 | 0.85 | 0.88 | 0.90 | 0.93 |
| WG240-sWG1-Sorbitol-30s-rep2 | 0.71 | 0.90 | 0.86 | 0.88 | 0.86 | 0.82 | 0.79 | 0.72 | 1.00 | 0.95 | 0.90 | 0.85 | 0.81 | 0.77 | 0.71 |
| WG241-sWG1-Sorbitol-1min-rep2 | 0.65 | 0.90 | 0.91 | 0.92 | 0.86 | 0.79 | 0.74 | 0.65 | 0.95 | 1.00 | 0.90 | 0.82 | 0.76 | 0.71 | 0.65 |
| WG242-sWG1-Sorbitol-2min-rep2 | 0.78 | 0.79 | 0.79 | 0.89 | 0.93 | 0.90 | 0.87 | 0.79 | 0.90 | 0.90 | 1.00 | 0.94 | 0.89 | 0.85 | 0.78 |
| WG243-sWG1-Sorbitol-3min-rep2 | 0.85 | 0.75 | 0.73 | 0.82 | 0.91 | 0.95 | 0.93 | 0.85 | 0.85 | 0.82 | 0.94 | 1.00 | 0.96 | 0.92 | 0.86 |
| WG244-sWG1-Sorbitol-5min-rep2 | 0.88 | 0.70 | 0.68 | 0.76 | 0.86 | 0.95 | 0.96 | 0.88 | 0.81 | 0.76 | 0.89 | 0.96 | 1.00 | 0.96 | 0.90 |
| WG245-sWG1-Sorbitol-10min-rep2 | 0.90 | 0.65 | 0.63 | 0.70 | 0.82 | 0.91 | 0.96 | 0.90 | 0.77 | 0.71 | 0.85 | 0.92 | 0.96 | 1.00 | 0.93 |
| WG246-sWG1-Sorbitol-30min-rep2 | 0.92 | 0.61 | 0.58 | 0.63 | 0.76 | 0.84 | 0.90 | 0.93 | 0.71 | 0.65 | 0.78 | 0.86 | 0.90 | 0.93 | 1.00 |

**B**

| | Cer3H4L2AmitS-WG223b-WG239_sWG1_Sorbitol_UT_-mtDNArDNA2UPCUP1_Ave | Cer3H4L2AmitS-WG224b-WG240_sWG1_Sorbitol_30s_-mtDNArDNA2UPCUP1_Ave | Cer3H4L2AmitS-WG225b-WG241_sWG1_Sorbitol_1min_-mtDNArDNA2UPCUP1_A | Cer3H4L2AmitS-WG226b-WG242_sWG1_Sorbitol_2min_-mtDNArDNA2UPCUP1_A | Cer3H4L2AmitS-WG227b-WG243_sWG1_Sorbitol_3min_-mtDNArDNA2UPCUP1_A | Cer3H4L2AmitS-WG228b-WG244_sWG1_Sorbitol_5min_-mtDNArDNA2UPCUP1_A | Cer3H4L2AmitS-WG229b-WG245_sWG1_Sorbitol_10min_-mtDNArDNA2UPCUP1_ | Cer3H4L2AmitS-WG246-sWG1-Sorbitol-30min-rep2_-mtDNArDNA2UPCUP1_scalec |
|---|---|---|---|---|---|---|---|---|
| Cer3H4L2AmitS-WG223b-WG239_sWG1_Sorbitol_UT_-mtDNArDNA2UPCUP1_Average | 1 | 0.69 | 0.63 | 0.74 | 0.84 | 0.88 | 0.91 | 0.93 |
| Cer3H4L2AmitS-WG224b-WG240_sWG1_Sorbitol_30s_-mtDNArDNA2UPCUP1_Average | 0.69 | 1 | 0.96 | 0.91 | 0.86 | 0.81 | 0.76 | 0.69 |
| Cer3H4L2AmitS-WG225b-WG241_sWG1_Sorbitol_1min_-mtDNArDNA2UPCUP1_Average | 0.63 | 0.96 | 1 | 0.93 | 0.84 | 0.77 | 0.72 | 0.64 |
| Cer3H4L2AmitS-WG226b-WG242_sWG1_Sorbitol_2min_-mtDNArDNA2UPCUP1_Average | 0.74 | 0.91 | 0.93 | 1 | 0.94 | 0.88 | 0.83 | 0.73 |
| Cer3H4L2AmitS-WG227b-WG243_sWG1_Sorbitol_3min_-mtDNArDNA2UPCUP1_Average | 0.84 | 0.86 | 0.84 | 0.94 | 1 | 0.96 | 0.92 | 0.84 |
| Cer3H4L2AmitS-WG228b-WG244_sWG1_Sorbitol_5min_-mtDNArDNA2UPCUP1_Average | 0.88 | 0.81 | 0.77 | 0.88 | 0.96 | 1 | 0.97 | 0.89 |
| Cer3H4L2AmitS-WG229b-WG245_sWG1_Sorbitol_10min_-mtDNArDNA2UPCUP1_Average | 0.91 | 0.76 | 0.72 | 0.83 | 0.92 | 0.97 | 1 | 0.93 |
| Cer3H4L2AmitS-WG246-sWG1-Sorbitol-30min-rep2_-mtDNArDNA2UPCUP1_scaled | 0.93 | 0.69 | 0.64 | 0.73 | 0.84 | 0.89 | 0.93 | 1 |

**Supplementary Figure 10. Reproducibility of data generated in this study. (A-B)** Grids report the pairwise Pearson correlation between the CC-seq-v2 Top2 activity osmotic shock libraries utilised in this study after first binning at 100 bp. Pearson values are colour coded from white (low, 0.5) to high (red, 1.0). In (**A**), individual biological replicates (rep1, rep2) are labelled in the DNA library names. Changes through time of osmotic shock are highlighted for replicate 1 (yellow box) and replicate 2 (green box). Inter-replicate correlations are highlighted in blue. The first replicate of the 30 minute time point was unsuccessful. In (B), equally weighted averages of untreated samples and each osmotic shock time point (except 30 minutes) were generated (as used in main figures) and compared.

| Strain | Genotype | Source |
|---|---|---|
| W303-1a | *Mata ade2-1 his3-11 leu2-3 trp1-1 ura3-1 can1-100* | Gift of Jon Baxter |
| W303-1a degron background control | *Mata ade2-1 his3-11 leu2-3 trp1-1 ura3-1 can1-100*<br>*UBR1::GAL1-10-Ubiquitin-M-LacI fragment-Myc-UBR1 (HIS3)*<br>*leu2-3::pCM244 (CMVp-tetR'-SSN6, LEU2) x3* | Gift of Jon Baxter |
| W303-1a *top2-td* | *Mata ade2-1 his3-11 leu2-3 trp1-1 ura3-1 can1-100*<br>*UBR1::GAL1-10-Ubiquitin-M-LacI fragment-Myc-UBR1 (HIS3)*<br>*leu2-3::pCM244 (CMVp-tetR'-SSN6, LEU2) x3*<br>*top2-td TOP2 5' upstream -100 to -1 replaced with kanMX-tTA (tetR-VP16)-tetO2 - Ub -DHFRts - Myc -linker)* | Gift of Jon Baxter |
| W303-1a *pdr1Δ::DBD-CYC8* | *Mata ade2-1 his3-11 leu2-3 trp1-1 ura3-1 can1-100*<br>*pdr1Δ::PDR1-DBD-CYC8::LEU2* | Gift of Jon Baxter |

**Supplementary Table 1. *S. cerevisiae* strains used in this study.** All strains are isogenic and from the W303 background. Top2 depletion experiments were performed in a derivative strain in which Top2 is tagged with a temperature-sensitive degron, and rapidly degraded by upregulation of galactose-induced *UBR1* expression[3]. The etoposide experiment was performed in a strain expressing a multidrug sensitivity allele described previously[4].

| Library | Type | Total reads (Millions) |
|---|---|---|
| WG204_YPD_rep1a | CC-seq-v2 | 11.13 |
| WG205_YPD_rep1b | CC-seq-v2 | 10.94 |
| WG206_YPD_rep1c | CC-seq-v2 | 11.91 |
| WG215_YPD_rep2 | CC-seq-v2 | 7.35 |
| WG134_wtdgb_Degron_rep1 | CC-seq-v2 | 2.24 |
| WG136_top2td_Degron_rep1 | CC-seq-v2 | 1.39 |
| WG223_UT_rep1 | CC-seq-v2 | 5.90 |
| WG224_OS-30s_rep1 | CC-seq-v2 | 6.52 |
| WG225_OS-1min_rep1 | CC-seq-v2 | 5.27 |
| WG226_OS-2min_rep1 | CC-seq-v2 | 6.88 |
| WG227_OS-3min_rep1 | CC-seq-v2 | 7.07 |
| WG228_OS-5min_rep1 | CC-seq-v2 | 6.24 |
| WG229_OS-10min_rep1 | CC-seq-v2 | 6.45 |
| WG239_UT_rep2 | CC-seq-v2 | 5.88 |
| WG240_OS-30s_rep2 | CC-seq-v2 | 5.69 |
| WG241_OS-1min_rep2 | CC-seq-v2 | 4.74 |
| WG242_OS-2min_rep2 | CC-seq-v2 | 4.28 |
| WG243_OS-3min_rep2 | CC-seq-v2 | 5.18 |
| WG244_OS-5min_rep2 | CC-seq-v2 | 5.54 |
| WG245_OS-10min_rep2 | CC-seq-v2 | 4.29 |
| WG246_OS-30min_rep2 | CC-seq-v2 | 4.39 |
| WG345_pdr1mut_UT_rep1 | CC-seq-v2 | 14.06 |
| WG346_pdr1mut_OS-2min_rep1 | CC-seq-v2 | 7.43 |
| WG347_pdr1mut_VP16-15min_rep1 | CC-seq-v2 | 9.82 |
| WG348_pdr1mut_VP16-15min_OS-2min_rep1 | CC-seq-v2 | 10.29 |
| WG349_pdr1mut_VP16-30min_rep1 | CC-seq-v2 | 4.54 |
| WG350_pdr1mut_VP16-30min_OS-2min_rep1 | CC-seq-v2 | 9.06 |
| WG170_YPR-0min_OS-2min_rep1 | CC-seq-v2 | 4.01 |
| WG171_YPR-0min_OS-2min_rep2 | CC-seq-v2 | 5.55 |
| WG172_YPG-30min_OS-2min_rep1 | CC-seq-v2 | 6.03 |
| WG173_YPG-30min_OS-2min_rep2 | CC-seq-v2 | 5.74 |
| WG174_YPG-60min_OS-2min_rep1 | CC-seq-v2 | 5.37 |
| WG175_YPG-60min_OS-2min_rep2 | CC-seq-v2 | 5.87 |
| WG176_YPG-90min_OS-2min_rep1 | CC-seq-v2 | 4.55 |
| WG177_YPG-90min_OS-2min_rep2 | CC-seq-v2 | 5.08 |
| WG178_YPR-90min_OS-2min_rep1 | CC-seq-v2 | 2.96 |
| WG179_YPR-90min_OS-2min_rep2 | CC-seq-v2 | 6.12 |
| WG199_YPR-0min_OS-2min_rep3 | CC-seq-v2 | 9.29 |
| WG200_YPG-30min_OS-2min_rep3 | CC-seq-v2 | 10.67 |
| WG201_YPG-60min_OS-2min_rep3 | CC-seq-v2 | 9.30 |
| WG202_YPG-90min_OS-2min_rep3 | CC-seq-v2 | 15.26 |

| Library | Type | Total reads (Millions) |
|---|---|---|
| WG203_YPR-90min_OS-2min_rep3 | CC-seq-v2 | 10.58 |
| WG208_YPR-0min_OS-2min_rep4 | CC-seq-v2 | 7.32 |
| WG209_YPR-30min_OS-2min_rep1 | CC-seq-v2 | 7.06 |
| WG210_YPR-60min_OS-2min_rep1 | CC-seq-v2 | 6.10 |
| WG211_YPR-90min_OS-2min_rep4 | CC-seq-v2 | 7.04 |
| WG212_YPG-30min_OS-2min_rep4 | CC-seq-v2 | 6.94 |
| WG213_YPG-60min_OS-2min_rep4 | CC-seq-v2 | 7.35 |
| WG214_YPG-90min_OS-2min_rep4 | CC-seq-v2 | 6.83 |
| WG216_YPR-0min_rep1 | CC-seq-v2 | 8.04 |
| WG217_YPR-30min_rep1 | CC-seq-v2 | 7.28 |
| WG218_YPR-60min_rep1 | CC-seq-v2 | 7.49 |
| WG219_YPR-90min_rep1 | CC-seq-v2 | 7.42 |
| WG220_YPG-30min_rep1 | CC-seq-v2 | 8.54 |
| WG221_YPG-60min_rep1 | CC-seq-v2 | 7.71 |
| WG222_YPG-90min_rep1 | CC-seq-v2 | 8.03 |
| WG184_YPD_rep1 | RNA-seq | 15.91 |
| WG188_YPR-0min_rep1 | RNA-seq | 20.87 |
| WG190_YPG-30min_rep1 | RNA-seq | 16.54 |
| WG192_YPG-60min_rep1 | RNA-seq | 15.75 |
| WG194_YPG-90min_rep1 | RNA-seq | 16.88 |
| WG185_YPD_rep2 | RNA-seq | 15.05 |
| WG189_YPR-0min_rep2 | RNA-seq | 16.89 |
| WG191_YPG-30min_rep2 | RNA-seq | 18.82 |
| WG193_YPG-60min_rep2 | RNA-seq | 18.99 |
| WG195_YPG-90min_rep2 | RNA-seq | 16.69 |
| WG300_UT_rep1 | MNase-seq | 15.27 |
| WG301_OS-30s_rep1 | MNase-seq | 14.28 |
| WG302_OS-1min_rep1 | MNase-seq | 11.42 |
| WG303_OS-2min_rep1 | MNase-seq | 11.83 |
| WG304_OS-3min_rep1 | MNase-seq | 11.49 |
| WG305_OS-5min_rep1 | MNase-seq | 12.17 |
| WG306_OS-10min_rep1 | MNase-seq | 11.08 |
| WG307_OS-30min_rep1 | MNase-seq | 11.06 |
| WG328_UT_rep2 | MNase-seq | 21.80 |
| WG329_OS-30s_rep2 | MNase-seq | 24.83 |
| WG330_OS-1min_rep2 | MNase-seq | 23.35 |
| WG331_OS-2min_rep2 | MNase-seq | 30.32 |
| WG332_OS-3min_rep2 | MNase-seq | 26.01 |
| WG333_OS-5min_rep2 | MNase-seq | 29.13 |
| WG334_OS-10min_rep2 | MNase-seq | 24.66 |
| WG335_OS-30min_rep2 | MNase-seq | 25.60 |

**Supplementary Table 2. CC-seq-v2, RNA-seq, and MNase-seq datasets generated in this study.** Individual CC-seq or RNA-seq libraries were prepared as indicated and sequenced using paired-end Illumina sequencing to the indicated mapped read depth.

| Data Type | Final Data | Component Libraries |
|---|---|---|
| CC-seq-v2 | YPD | WG204, WG205, WG206, WG215, WG223, WG239 |
| CC-seq-v2 | UT | WG223, WG239 |
| CC-seq-v2 | OS-30s | WG224, WG240 |
| CC-seq-v2 | OS-1min | WG225, WG241 |
| CC-seq-v2 | OS-2min | WG226, WG242 |
| CC-seq-v2 | OS-3min | WG227, WG243 |
| CC-seq-v2 | OS-5min | WG228, WG244 |
| CC-seq-v2 | OS-10min | WG229, WG245 |
| CC-seq-v2 | OS-30min | WG246 |
| CC-seq-v2 | AllYPR | WG216, WG217, WG218, WG219 |
| CC-seq-v2 | AllYPG | WG220, WG221, WG222 |
| CC-seq-v2 | AllYPR_OS-2min | WG170, WG171, WG178, WG179, WG199, WG203, WG208, WG209, WG210, WG211 |
| CC-seq-v2 | AllYPG_OS-2min | WG172, WG173, WG174, WG175, WG176, WG177, WG200, WG201, WG202, WG212, WG213, WG21 |
| CC-seq-v2 | wtdgb_Degron | WG134 |
| CC-seq-v2 | top2td_Degron | WG136 |
| CC-seq-v2 | pdr1mut_UT | WG345 |
| CC-seq-v2 | pdr1mut_OS-2min | WG346 |
| CC-seq-v2 | pdr1mut_VP16-15min | WG347 |
| CC-seq-v2 | pdr1mut_VP16-15min_OS-2min | WG348 |
| CC-seq-v2 | pdr1mut_VP16-30min | WG349 |
| CC-seq-v2 | pdr1mut_VP16-30min_OS-2min | WG350 |
| RNA-seq | YPD | WG184, WG185 |
| RNA-seq | YPR-0min | WG188, WG189 |
| RNA-seq | YPG-30min | WG190, WG191 |
| RNA-seq | YPG-60min | WG192, WG193 |
| RNA-seq | YPG-90min | WG194, WG195 |
| MNase-seq | OS-UT | WG300, WG328 |
| MNase-seq | OS-30s | WG301, WG329 |
| MNase-seq | OS-1min | WG302, WG330 |
| MNase-seq | OS-2min | WG303, WG331 |
| MNase-seq | OS-3min | WG304, WG332 |
| MNase-seq | OS-5min | WG305, WG333 |
| MNase-seq | OS-10min | WG306, WG334 |
| MNase-seq | OS-30min | WG307, WG335 |

**Supplementary Table 3. CC-seq-v2 library averages used in this study.** Component CC-seq-v2 datasets were averaged together with equal weighting to generate the final datasets indicated.

## Supplementary References:

1. Gittens WH, Johnson DJ, Allison RM, Cooper TJ, Thomas H, Neale MJ. A nucleotide resolution map of Top2-linked DNA breaks in the yeast and human genome. *Nat Commun* **10**, 4846 (2019).

2. Guo MS, Kawamura R, Littlehale ML, Marko JF, Laub MT. High-resolution, genome-wide mapping of positive supercoiling in chromosomes. *eLife* **10**, e67236 (2021).

3. Minchell NE, Keszthelyi A, Baxter J. Cohesin Causes Replicative DNA Damage by Trapping DNA Topological Stress. *Mol Cell* **78**, 739-751.e738 (2020).

4. Stepanov A, Nitiss KC, Neale G, Nitiss JL. Enhancing drug accumulation in Saccharomyces cerevisiae by repression of pleiotropic drug resistance genes with chimeric transcription repressors. *Mol Pharmacol* **74**, 423-431 (2008).
